# Overexpression of *ZmDHN15* Enhances Cold Tolerance in Yeast and Arabidopsis

**DOI:** 10.3390/ijms24010480

**Published:** 2022-12-28

**Authors:** Nannan Chen, Xuhong Fan, Chunlai Wang, Peng Jiao, Zhenzhong Jiang, Yiyong Ma, Shuyan Guan, Siyan Liu

**Affiliations:** 1 College of Life Sciences, Jilin Agricultural University, Changchun 130118, China; 2 Joint International Research Laboratory of Modern Agricultural Technology, Ministry of Education, Jilin Agricultural University, Changchun 130118, China; 3 Jilin Academy of Agricultural Sciences, Changchun 130118, China; 4 College of Agronomy, Jilin Agricultural University, Changchun 130118, China

**Keywords:** *ZmDHN15* gene, maize, dehydrin, cold stress

## Abstract

Maize (*Zea mays* L.) originates from the subtropical region and is a warm-loving crop affected by low-temperature stress. Dehydrin (DHN) protein, a member of the Group 2 LEA (late embryogenesis abundant proteins) family, plays an important role in plant abiotic stress. In this study, five maize DHN genes were screened based on the previous transcriptome sequencing data in our laboratory, and we performed sequence analysis and promoter analysis on these five DHN genes. The results showed that the promoter region has many cis-acting elements related to cold stress. The significantly upregulated *ZmDHN15* gene has been further screened by expression pattern analysis. The subcellular localization results show that *ZmDHN15* fusion protein is localized in the cytoplasm. To verify the role of *ZmDHN15* in cold stress, we overexpressed *ZmDHN15* in yeast and Arabidopsis. We found that the expression of *ZmDHN15* can significantly improve the cold resistance of yeast. Under cold stress, *ZmDHN15*-overexpressing Arabidopsis showed lower MDA content, lower relative electrolyte leakage, and less ROS (reactive oxygen species) when compared to wild-type plants, as well as higher seed germination rate, seedling survival rate, and chlorophyll content. Furthermore, analysis of the expression patterns of ROS-associated marker genes and cold-response-related genes indicated that *ZmDHN15* genes play an important role in the expression of these genes. In conclusion, the overexpression of the *ZmDHN15* gene can effectively improve the tolerance to cold stress in yeast and Arabidopsis. This study is important for maize germplasm innovation and the genetic improvement of crops.

## 1. Introduction

Maize (*Zea mays* L.) is an important food crop and industrial raw material crop, ranking first in the world’s total food production [1]. Maize originates from the subtropical zone and is a thermophilic crop affected by low-temperature stress [2]. The germination and seedling stages of maize in the northern spring sowing area are extremely vulnerable to low temperatures [3]. Therefore, mining the cold-resistance genes of maize and studying their mechanisms have become a hot issue. The impact of low temperature on corn is divided into chilling damage (below 15 °C) and freezing damage (below 0 °C) [4]. Chilling damage can destroy the metabolic balance of water, inhibit respiration and photosynthesis, change the microstructure of cells, aggravate protein decomposition and metabolism, produce toxic substances, and disrupt hormone synthesis balance [5,6,7,8,9,10,11]. Furthermore, like all other stresses, cold stress causes oxidative stress due to an imbalance between the production of reactive oxygen species (ROS) and the ability of enzymatic and non-enzymatic antioxidant defense systems to process ROS [12].

Late-embryogenesis-abundant proteins (LEA proteins) are a class of proteins that play important roles under plant stress [13]. They were first identified during cotton embryo development and abundantly accumulated during late seed maturation [14]. Most LEA proteins are rich in hydrophilic amino acids, such as glycine, lysine, and histidine, and have high hydrophilicity and thermal stability [15,16]. LEA proteins mainly protect desiccation during seed dehydration or vegetative tissues under stress conditions by acting as cell-dehydration protectors [17]. LEA proteins can be divided into different subfamilies according to their domains Among them, dehydrin (DHN) is a member of the Group 2 LEA [8,18]. Dehydrin is a kind of hydrophilic protein that widely exists in plants and is generally composed of 82–575 amino acids. The molecular weight of different dehydrins varies greatly, ranging from 9 to 200 kDa [13,19]. An important structural feature of dehydrin is that it has three highly conserved regions. The K domain is a conserved segment of dehydrin rich in lysine, and its sequence is usually XKXGXX(D/E)KIK(D/E)KXPG. The sequence of the Y domain is usually D(D/E/Q)(Y/H/F)GNP. The S domain has four to six tandem serines, and the conserved sequence is SSSSSED [15,16,18]. Dehydrin can be divided into five subclasses according to the presence and order of K, Y, and S domains, namely Kn, SKn, KnS, YnKn, and YnSKn [20]. Dehydrin is induced by various abiotic stresses, such as low temperature, drought, salinity, hormones, and osmotic pressure [21]. Dehydrin function primarily as molecular shields, and their intrinsic disorder is required for them to be an effective cryoprotectant [22,23]. Generally, it reduces the stress of cells in low temperature, dehydration, etc., by protecting enzyme activity, reducing membrane phase-transition temperature, and binding heavy metal ions [24,25,26,27]. In recent years, previous studies have found that dehydrin genes are widely used in plant stress resistance. For example, the overexpression of Prunus mume dehydrin genes *PmLEA10*, *PmLEA19*, *PmLEA20,* and *PmLEA29* in tobacco improves cold resistance [23]. The wheat dehydrin *wzy1-2* gene can respond to cold stress [28]. The heterologous expression of *CsLEA1* increased the tolerance of *Escherichia coli* and yeast to cold stress [29]. Low temperatures induced the expression of *PcLEA14,* and low-temperature stress tolerance in Arabidopsis was improved after its overexpression [30]. Pepper *CaDHN3* may play an important role in regulating the relative osmotic stress response of plants through the ROS signaling pathway [31]. *SiLEA4* is important in conferring microorganisms and plants’ low-temperature-stress resistance [32]. At the same time, a large number of studies have shown that dehydrin genes plays a crucial role in maize defense under different abiotic stress conditions. *ZmDHN13* positively regulates transgenic yeast and tobacco tolerance to copper stress by binding metals and reducing the formation of ROS [33]. The expression of *ZmDHN2b* in maize was induced by cold, and the overexpression of *ZmDHN2b* confered cold tolerance on tobacco [34]. The overexpression of *ZmDHN11* could enhance transgenic yeast and tobacco tolerance to osmotic stress [35].

Although information on dehydrin genes from various plant species is available, however, there are still many unknown mechanisms for the regulation, biochemical function, and physiological mechanism of DHN genes under cold stress in maize to be analyzed. In this study, based on previous transcriptome sequencing data in the laboratory, five maize DHN genes were identified using bioinformatics methods, and their structural features, promoter sequences, and expression patterns of these genes under cold stress. In addition, we investigated the overexpression of *ZmDHN15* in yeast and Arabidopsis, confirming the role of *ZmDHN15* in enhancing cold tolerance in eukaryotes and plants. This study provides a theoretical basis for studying cold-tolerance mechanisms in Poaceae.

## 2. Results

### 2.1. Bioinformatics Analysis of Maize Dehydrin Gene

**Figure 1:**
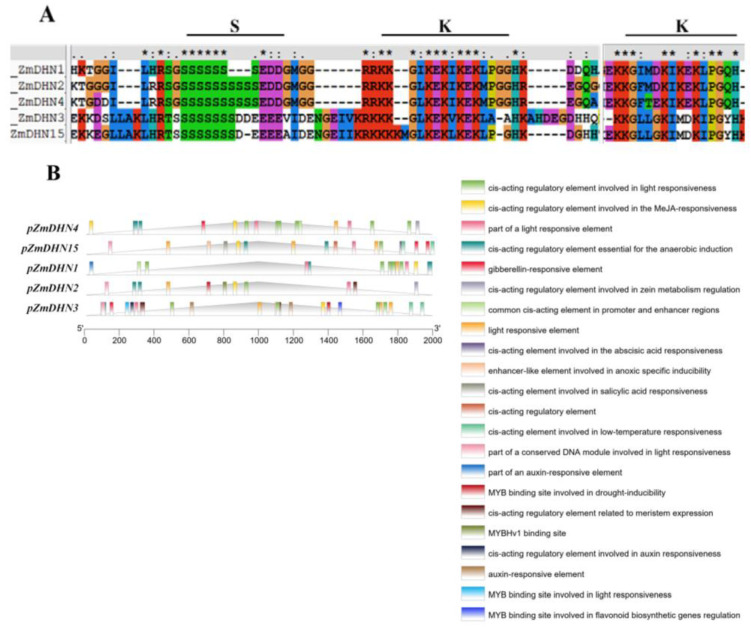
Bioinformatics analysis of maize dehydrin gene. (**A**) Homology alignment of the conserved sequences of five dehydrin in maize. (**B**) Analysis of cis-acting elements in the promoter sequences of five dehydrin genes. The “*” indicate agreement between the five conserved amino acid sequences of dehydrogenase in maize.

The amino acid sequence homology alignment of five dehydrin genes in maize showed that all five dehydrin genes had S-type and Y-type conserved sequences (Figure 1A). The 2000 bp upstream of the initiation codons (ATG) of these five dehydrin genes was used as the promoter sequence, and the cis-acting element analysis was carried out on the promoter sequence. The results showed that the promoter contained multiple functional elements related to the response to cold, light, hormone, and non-stress stress (Figure 1B).

### 2.2. Analysis of the Expression Pattern of the Maize Dehydrin Gene under Cold Stress

**Figure 2:**
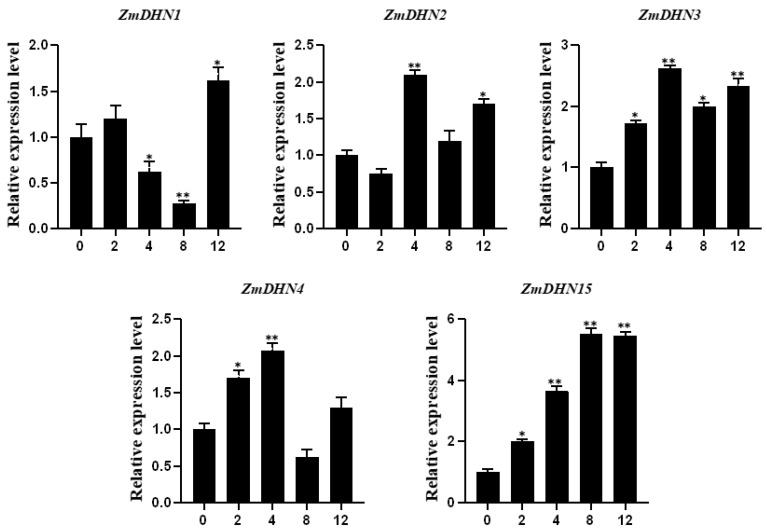
qRT-PCR analysis of the expression profiles of the maize dehydrin gene family. The expression level was normalized to that of maize *ZmACTIN1.* 0: cold stress 0 h, 2: cold stress 2 h, 4: cold stress 4 h, 8: cold stress 8 h, 12: cold stress 12 h. Data were expressed as the mean of triplicate values, and the error represented the SD. Asterisks indicate statistically significant differences: *p* < 0.05 (*) and *p* < 0.01 (**).

In order to screen the functional genes of cold resistance among the above five maize dehydrin genes. qRT-PCR results showed that *ZmDHN15* was significantly upregulated among these five genes (Figure 2). In other words, *ZmDHN15* may play an important role in the response of maize to cold stress.

### 2.3. Subcellular Localization of ZmDHN15 Protein

**Figure 3:**
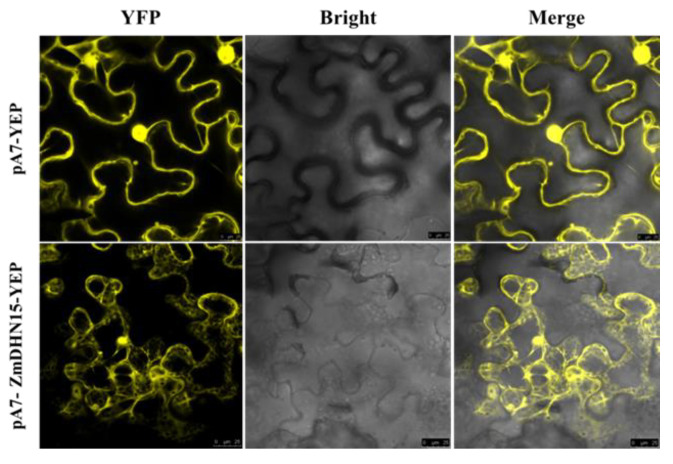
Subcellular localization of *ZmDHN15* protein. The positive controls pA7-YEP and pA7-ZmDHN15-YEP were both expressed in tobacco epidermal cells, showing the cytoplasmic localization of ZmDHN15.

Understanding the subcellular localization of gene expression products is important for the functional analysis of genes. *ZmDHN15* CDS (coding sequences) was cloned into the transient expression vector pA7-YEP. The subcellular localization of *ZmDHN15* protein was observed under a confocal microscope using the yellow fluorescence properties of YEP. The result shows that *ZmDHN15* protein is localized in the cytoplasm of tobacco epidermal cells (Figure 3).

### 2.4. Low-Temperature-Tolerance Assays of Yeast Transformants

**Figure 4:**
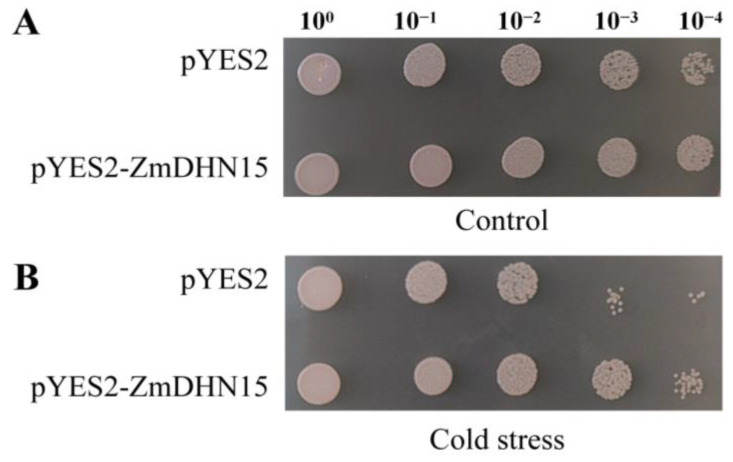
Overexpression of *ZmDHN15* enhances tolerance to cold stress in transformant yeast (*INVSc1*). (**A**) The transformant yeast was grown in 30 °C, 48 h. (**B**) The transformant yeast was grown in 4 °C, 48 h.

In order to determine the effect of the *ZmDHN15* protein on the survival rate of yeast recombinants under osmotic stress, we investigated the plaque growth of yeast cell lines transformed with a pYES2-ZmDHN15 vector and a control strain containing empty vector (pYES2) under cold stress at 4 °C. Under optimal conditions, the growth of the transformed yeast and the control yeast was not significantly different. Under the cold-stress condition of 4 °C, the transformed yeast showed stronger growth ability than the control group (Figure 4). Therefore, we believe that overexpression of *ZmDHN15* can improve the tolerance of yeast transformants.

### 2.5. Generation of Transgenic Plants and Molecular Identification

**Figure 5:**
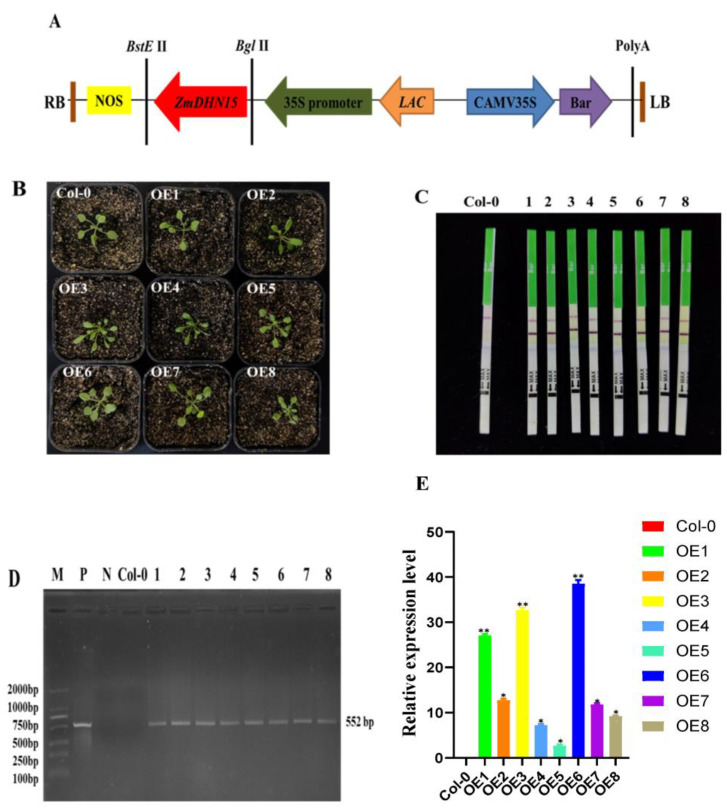
Identification of Arabidopsis overexpressing the *ZmDHN15* gene. (**A**) pCAMBIA3301- ZmDHN15 vector map. (**B**) Untransformed plant and transgenic plants. (**C**) Test strip detection. Col-0: Untransformed plant, 1–8: turn pCAMBIA3301-ZmDHN15 positive plants. (**D**) PCR detection of bar gene in transgenic *Arabidopsis thaliana*, M: DNA Marker DL 2000, P: pCAMBIA3301-ZmDHN15 recombinant plasmid, N: Negative control, Col-0: Untransformed plant, 1–8: turn pCAMBIA3301-ZmDHN15 positive plants. (**E**) qRT-PCR validation of transgenic *Arabidopsis thaliana*. Data were expressed as the mean of triplicate values, and the error represented the SD. Asterisks indicate statistically significant differences: *p* < 0.05 (*) and *p* < 0.01 (**).

The recombinant plasmid pCAMBIA3301-ZmDHN15 was constructed and introduced into wild-type Arabidopsis Col-0 lines (non-transgenic plant lines); *ZmDHN15* was overexpressed in Arabidopsis, and eight transgenic lines were generated (Figure 5A). Molecular detection was conducted on T3 transgenic plants. Three homozygous lines (OE1, OE3, and OE6) with the highest expression level were selected for follow-up experiments (Figure 5C–E).

### 2.6. Overexpression of ZmDHN15 Enhances Cold Resistance in Transgenic Arabidopsis

**Figure 6:**
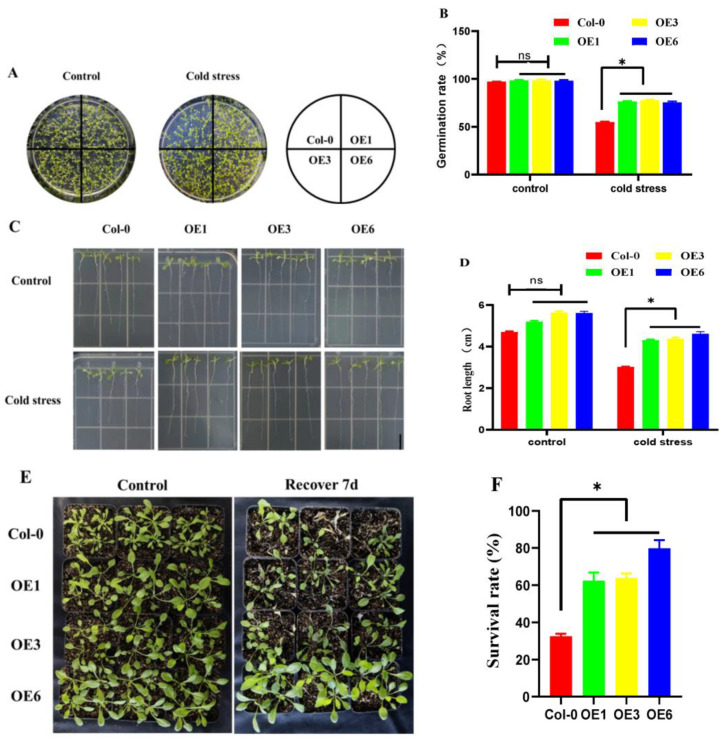
Phenotypes of the *ZmDHN15*-overexpressing transgenic plants and wild-type controls in Arabidopsis under cold stress. (**A**,**B**) The germination rate of *ZmDHN15* transgenic Arabidopsis with cold stress. (**C**,**D**) Root length statistics of transgenic *Arabidopsis thaliana ZmDHN15* under cold stress. Scale bars represent 1 cm. (**E**) Three-week-old Col-0 and OE lines recovered to 7 d phenotype after 24 h treatment at 4 °C. (**F**) Statistics on the survival rate of three-week-old Col-0 and OE lines treated at 4 °C for 24 h and recovered for 7 d. Data were expressed as the mean of triplicate values, and the error represented the SD. Asterisks indicate statistically significant differences: Non-significant (ns), *p* < 0.05 (*).

In order to investigate the function of *ZmDHN15*, cold-tolerance identification was performed on the identified OE lines (OE1, OE3, and OE6) and Col-0 lines during germination. Under normal conditions, the seed germination rate of Col-0 lines was similar to that of OE lines (overexpressing plant lines) but significantly decreased at 4 °C (Figure 6A,B). Furthermore, we found that the growth potential of Col-0 seedlings was weaker than that of OE strains under normal conditions. However, the growth potential of Col-0 seedlings was more affected than OE strains with the decrease of temperature (Figure 6C,D). These results suggest that OE lines of *ZmDHN15* in Arabidopsis can enhance the germination and root growth ability of plants under cold-stress conditions.

In order to further understand the response of transgenic Arabidopsis under cold stress, the plants of the Col-0 and OE lines were tested for cold tolerance. When exposed to 4 °C for 24 h and allowed to recover at room temperature for 7 d, the transgenic plants showed higher survival rates than Col-0 lines (Figure 6E,F). These results suggest that the OE lines of *ZmDHN15* in Arabidopsis improve the cold tolerance of plants.

### 2.7. Phenotypic Characterization of Overexpressed ZmDHN15 at the Mature Stage

**Figure 7:**
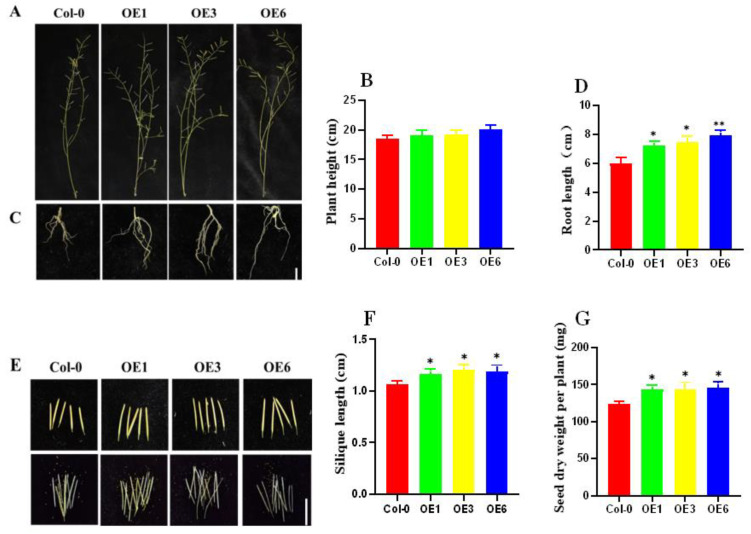
Agronomic traits of *ZmDHN15*-overexpressing transgenic plants under cold stress. (**A**,**B**) Plant height. (**C**,**D**) Root length. (**E**,**F**) Silique length. (**G**) Seed dry weight per plant. Scale bars represent 1 cm. Data were expressed as the mean of triplicate values, and the error represented the SD. Asterisks indicate statistically significant differences: *p* < 0.05 (*) and *p* < 0.01 (**).

The further phenotypic identification of mature plants of Col-0 and OE lines was performed. We observed no significant difference in the plant height of the OE lines compared with wild-type Col-0 plants (Figure 7A,B); however, the root lengths of the OE lines were significantly different (Figure 7C,D). In addition, the silique length and seed dry weight per plant were also significantly higher in the OE line than in the wild-type Col-0 plant (Figure 7E–G). These phenomena suggest that the OE of *ZmDHN15* in *Arabidopsis thaliana* improves the cold resistance of plants that are subjected to cold stress at the seedling stage and reduced the damage to plants caused by low temperature. Therefore, the introduction of this gene can effectively affect the yield of plants.

### 2.8. Overexpression of ZmDHN15 Reduces the ROS Accumulation

**Figure 8:**
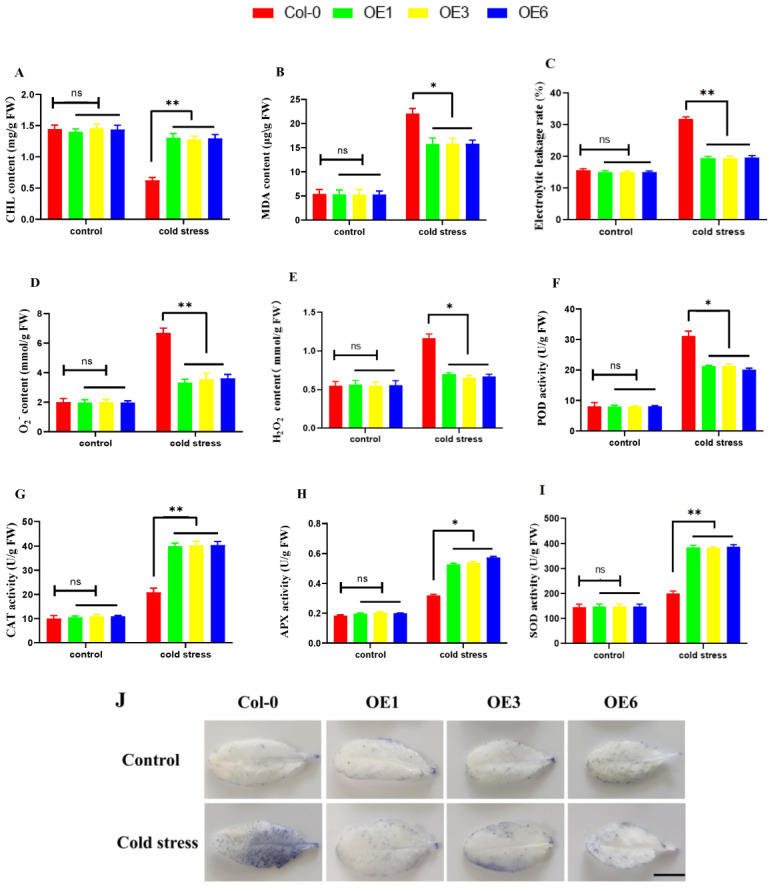
Reactive oxygen species staining and physiological indices in *ZmDHN15* transgenic Arabidopsis: (**A**) Chlorophyll (CHL) content. (**B**) Malondialdehyde (MDA) content. (**C**) Electrolytic leakage (EL) rate. (**D**) Superoxide radical (O_2_^−^) content in leaves. (**E**) Hydrongen peroxide (H_2_O_2_) content in leaves. (**F**) Analysis of peroxidase dismutase (POD) activity. (**G**) Analysis of catalase (CAT) activity. (**H**) Analysis of ascorbate peroxidase dismutase (APX) activity. (**I**) Analysis of superoxide dismutase (SOD) activity. (**J**) NBT staining was performed on Col−0 and transgenic lines subjected to 24 h cold stress to monitor ROS production in leaves under cold stress. Control: group not under cold stress, cold stress: low−temperature stress at 4 °C for 24 h. Scale bars represent 1 cm. Data were expressed as the mean of triplicate values, and the error represented the SD. Asterisks indicate statistically significant differences: Non−significant (ns), *p* < 0.05 (*) and *p* < 0.01 (**).

In order to study the effect of *ZmDHN15* on plant physiology and biochemistry, the chlorophyll (CHL), malondialdehyde (MDA) contents, and electrolytic leakage rate (EL) of Col-0 plants and OE lines were determined. Under cold stress, the CHL content of OE lines was significantly increased, and the content of MDA and the EL rate was significantly decreased compared with Col-0 lines (Figure 8A–C). Cold conditions usually cause oxidative damage to plants. In order to explore whether *ZmDHN15* can reduce ROS accumulation, antioxidant enzyme activities, peroxidase activities, and the contents of H_2_O_2_ and O_2_^−^ were measured. O_2_^−^ expression levels were detected by the NBT (Nitro-Blue Tetrazolium Chloride) staining technique. Under normal conditions, there were no significant differences in H_2_O_2_ and O_2_^−^ contents, antioxidant enzyme activities, and peroxidase activities between Col-0 and OE lines. After cold stress, OE lines showed lower H_2_O_2_ and O_2_^−^ contents and higher enzymatic activity (Figure 8D–I). The NBT staining results were consistent with O_2_^−^ assay results (Figure 8J). Under cold-stress conditions, all OE lines exhibited less damage and accumulated less ROS than Col-0 lines. Therefore, it is speculated that the expression of *ZmDHN15* may reduce ROS accumulation by increasing antioxidant enzymes and peroxidase activities in leaves.

**Figure 9:**
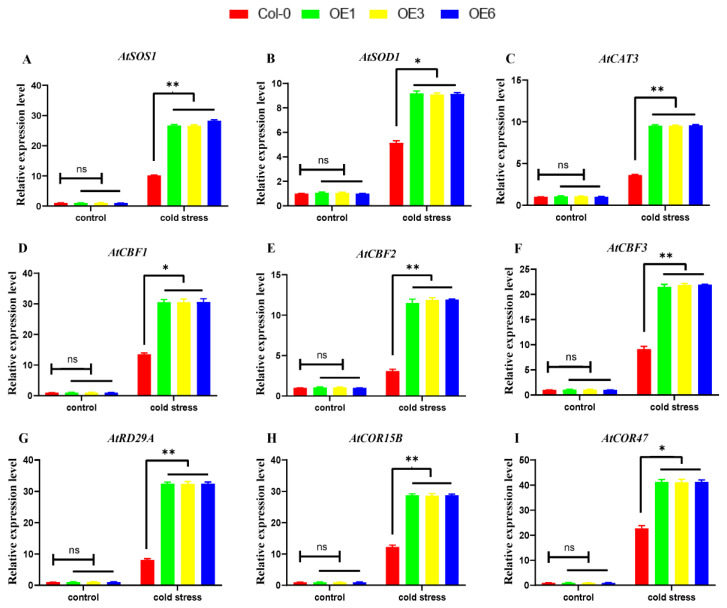
Analysis of ROS-related marker genes and cold-response-related genes in transgenic plants. (**A**–**C**) ROS-related marker genes expression analysis. (**D**–**I**) Cold-response-related genes expression analysis. Control: before the low-temperature stress at 4 ℃, cold stress: low-temperature stress at 4 ℃ for 24 h. The expression level was normalized to that of *AtACTIN1*. Data were expressed as the mean of triplicate values, and the error represented the SD. Asterisks indicate statistically significant differences: Non- significant (ns), *p* < 0.05 (*) and *p* < 0.01 (**).

The expression patterns of ROS-related marker genes and cold-response-related genes were investigated to further investigate the mechanism of *ZmDHN15.* The selected nine marker genes were expressed in Col-0 and OE lines under cold stress (Figure 9). The results show that the *ZmDHN15* gene plays an important role in the increased expression of nine marker genes in transgenic plants. *ZmDHN15* is a positive regulator of plant cold resistance and may enhance cold resistance through a CBF-dependent pathway in Arabidopsis. Notably, compared with Col-0 plants, all OE lines showed a minimal injury with a similar trend in physiological indices and the expression of genes related to cold resistance.

## 3. Discussion

Dehydrin (DHN) proteins have irregular structures that can effectively resist freezing. At low intracellular water potential, DHN proteins adsorb water molecules and act as osmoregulators [36]. DHN proteins are closely related to plant growth and development, except for their roles in stress response and distribution in different plant organs [37]. In this study, we identified five genes in the maize dehydrin gene family by bioinformatics and verified the biological function of *ZmDHN15* under cold stress by a series of experiments. Multiple sequence alignment results showed that the five DHN proteins contained highly conserved DHN domains and different distributions of K and S segments. The findings were also confirmed in previous studies [20,38]. The promoter sequences of these five genes all have cold-responsive cis-acting elements. qPT-PCR was performed on these five genes under cold stress, and the results showed that *ZmDHN15* was significantly upregulated under cold stress. Previous studies have shown that the homologous or heterologous expression of the DHN gene plays an important role in plant abiotic stress. Ju et al. heterologously expressed the maize *ZmDHN11* gene in yeast and tobacco, which enhanced the tolerance of transgenic yeast and tobacco to osmotic stress [35]. Zhang et al. overexpressed pepper *CaDHN4* in *Arabidopsis thaliana*, enhancing salt- and cold-stress tolerance [39]. In this study, the subcellular localization results showed that the ZmDHN15 protein was localized in the cytoplasm. The finding was consistent with previous reports [40]. The eukaryotic *Saccharomyces cerevisiae* is an ideal model organism for studying the stress-resistance function of genes, which has the advantages of speed and accuracy [39,40]. Its cell structure is more suitable for studying eukaryotic gene function. In this study, the stress-resistance function of *ZmDHN15* was investigated using the *Saccharomyces cerevisiae* expression system. The results showed that the tolerance of *ZmDHN15* transgenic yeast to cold stress was significantly improved. *Arabidopsis thaliana* has the advantages of a small plant size, more fruit, short life cycle, simple genome, and easy genetic manipulation [41,42] and is usually considered a model organism in various biological research disciplines based on plant material. In order to investigate the function of *ZmDHN15*, we generated transgenic Arabidopsis constitutively overexpressing *ZmDHN15*. We observed that, under cold-stress conditions, the germination rate and root length of OE lines were also higher than those of Col-0 lines. The measurement of some physiological and biochemical indicators showed that, under cold stress, the CHL content of the OE lines was significantly increased, and the content of MDA and EL rate was significantly decreased compared with the Col-0 lines. The results show that the overexpression of *ZmDHN15* can significantly enhance the cold resistance of yeast and Arabidopsis.

ROS is important for species homeostasis and signal transduction [31]. The production and scavenging of ROS in plants are in a state of dynamic equilibrium under normal conditions [4,43]. More ROS, including superoxide anion (O_2_^−^) and hydrogen peroxide (H_2_O_2_), etc., will be accumulated when plants are under abiotic stress, which may cause oxidative damage to biomolecules in plants [44]. Studies have shown that increasing the expression of ROS scavenging-related genes can improve plant stress tolerance [42]. Mbukeni Nkomo et al. found that higher levels of H_2_O_2_ could aggravate plant oxidative damage [45]. Wang et al. found that salt stress would decrease the photosynthetic efficiency of Arabidopsis, damage the membrane, and accumulate more ROS [46]. Although ROS molecules mediate plant growth by activating many stress-related genes [31,46], the effect of DHN genes on ROS accumulation is unclear. In this study, the activities of CAT, SOD, POD, and APX were measured under cold-stress conditions, and the ROS accumulation in Arabidopsis leaves was also analyzed to explore the effect of the *ZmDHN15* gene on the scavenging mechanism of ROS. Compared with Col-0 lines, the SOD, CAT and APX activities of OE lines increased, POD activities decreased, the accumulation of H_2_O_2_ and O_2_^−^ decreased, and the NBT staining results became light. These results indicate that the *ZmDHN15* gene can reduce the ROS accumulation in vivo, thereby enhancing the tolerance of plants to cold stress. We also investigated ROS-related marker genes [10,47]. The expression levels of the selected three marker genes were induced in Col-0 and OE lines under cold stress. We analyzed the expression of several widely reported cold-related genes [2] (*AtRD29A*, *AtCOR15B*, and *AtCOR47*) in Col-0 and OE lines to further analyze the function of *ZmDHN15* in plant cold resistance. The results showed that all genes were upregulated under low-temperature stress, and the changes in OE lines were more drastic than in Col-0 lines. The expression trends of physiological indicators and cold-resistance-related genes were similar. The above results indicate that *ZmDHN15* is a positive regulator of plant cold resistance. According to a previous analysis, the *ZmDHN15* promoter sequence has a CRT/DRE response element, indicating that the expression of *ZmDHN15* may be regulated by CBF/DREB transcription factors in response to ICE-CBF-COR cascading pathways to improve plant cold resistance [6,48,49,50,51]. Furthermore, the expression patterns of CBF1, CBF2, and CBF3 in Col-0 and OE lines showed that all genes were expressed under low-temperature stress. Therefore, it is predicted that cold resistance may be enhanced through a CBF-dependent pathway in Arabidopsis.

## 4. Materials and Methods

### 4.1. Plant Materials and Growth Conditions

In this study, the wild-type Col-0 of *Arabidopsis thaliana* and the maize inbred line H8069 provided by the Plant Biology Center of Jilin Agricultural University were used as materials. Maize plants were grown under long-day conditions (light 16 h, dark 8 h) at 24 °C and relative humidity of 46%, and transgenic Arabidopsis plants were grown at 24 °C and relative humidity of 65% under long-day conditions.

### 4.2. Screening of Maize Dehydrin Gene under Cold Stress

According to the early transcriptome sequencing data in the laboratory (not disclosed), five genes (*ZmDHN1*: GRMZM2G079440, *ZmDHN2*: GRMZM2G098750, *ZmDHN3*: GRMZM2G373522, *ZmDHN4*: GRMZM2G052364, and *ZmDHN15*: GRMZM2G147014) in maize DHN gene family were screened. The amino acid sequences of five genes in the maize DHN gene family were downloaded from the National Center for Biotechnology Information (NCBI) and maizeGDB (https://www.maizegdb.org/) (accessed on 12 March 2021) libraries for amino acid sequence homology alignment analysis. Meanwhile, Plant CARE (http://bioinformatics.psb.ugent.be/webtools/plantcare/html/) (accessed on 13 March 2021) was used to analyze the promoter sequences of different abiotic-stress-related cis-elements [52]. 

The corn inbred line H8069 growing to the four-leaf stage was treated with a low temperature. Samples were collected at 0 h and 12 h of treatment, immediately frozen in liquid nitrogen, and fully ground. Then, total RNA was extracted by the Trizol method and reverse-transcribed into cDNA using a reverse transcription kit. The qPCR amplification was performed on the five genes of the DHN as mentioned above gene family using the SYBR Green qRT-PCR SuperMix instructions. Furthermore, 2^−ΔΔCT^ was used for data processing with three replicates per sample. All primers used in the experiments are listed in Supplementary Table S1. 

### 4.3. Subcellular Localization of ZmDHN15

The *ZmDHN15* CDS without the stop codon was amplified by Vector Cloning Kit and cloned into the plant expression vector pA7-YEP (restriction sites: *Xho* I and Hind III) to construct the recombinant plasmid pYES2-ZmDHN15. The recombinant plasmid was expressed in tobacco leaves by the Agrobacterium-mediated method, and the expression of YEP was observed by fluorescence confocal microscopy with the empty vector pA7-YEP as the control.

### 4.4. Expression of ZmDHN15 in Saccharomyces Cerevisiae INVSc1

The *ZmDHN15* cloning vector was inserted into the yeast expression vector pYES2 (restriction sites: *Hind* III and *Bam* HI) to construct the recombinant plasmid pYES2-ZmDHN15 and transform *Saccharomyces cerevisiae INVSc1*. The pYES2 empty vector was transformed as a negative control. The transformation was verified by bacterial liquid PCR results [53].

### 4.5. Low-Temperature Tolerance Assay of Yeast Transformants

*Saccharomyces cerevisiae INVSc1* carrying the pYES2-ZmDHN15 recombinant plasmid and confirmed positive empty vector pYES2 were induced with galactose and then undiluted and diluted 10^−1^, 10^−2^, 10^−3^, and 10^−4^ times, respectively. Then, 2 μL samples were taken and spotted on an S-U (containing 2% glucose) solid medium. After culturing at 4 °C for 48 h, the growth differences between the two strains were analyzed. The two strains cultured at 30 °C for 48 h were used as the control.

### 4.6. Generation of Transgenic Plants and Phenotypic Analysis

The pCAMBIA-3301-ZmDHN15 vector was constructed by inserting the *ZmDHN15* gene cloning vector into the plant high expression vector pCAMBIA-3301(enzyme cleavage sites: *Bgl* II and *BstE* II). The product was introduced into wild-type Col-0 lines by the Agrobacterium-mediated floral dipping method to obtain transgenic lines. Homozygous plants were screened with 1/2 MS solid medium, and the subculture was continued. For the T3 generation, three high expressing lines were selected for further study. All primers used in the experiments are listed in Supplementary Table S1.

Col-0 and three highly expressed T3 generation transgenic Arabidopsis seeds were sterilized by 70% and 5% in turn, then sown on 1/2 MS medium and placed in a 4 °C incubator under long-day light conditions (light for 16 h). The control group was grown under long-day conditions (light 16 h, dark 8 h) in a 24 °C incubator. After 14 days, the germination of seeds was recorded, and the germination rate and root length of seedlings were counted.

In order to further understand the response of transgenic Arabidopsis to cold stress, three-week-old Col-0 and three high-expressing T3 transgenic Arabidopsis lines were subjected to cold stress at 4 °C for 24 h, followed by recovery at room temperature for 7 d. The phenotypic changes of Arabidopsis plants were photographed, and the survival rate was counted during the treatment period.

### 4.7. Determination of Chlorophyll, Malondialdehyde, H_2_O_2_, and O_2_^−^ Content and Antioxidative Enzyme Activity

The three-week-old Col-0 and three highly expressed T3 transgenic Arabidopsis lines were treated with cold stress at 4 °C for 24 h, and the following physiological and biochemical indicators were measured before and after cold stress: chlorophyll, electrolytic leakage (EL) rate, MDA, H_2_O_2_, and O_2_^−^. The contents were detected by the method proposed by Jing et al. [54,55], and the activities of SOD, POD, CAT, and APX were determined by Yu and Zhang et al. [39,56,57]. Three replicates were performed for each sample.

### 4.8. NBT (Nitro-Blue Tetrazolium Chloride) Staining Assay

Three-week-old Col-0 and three high-expressing T3 transgenic Arabidopsis lines were treated with cold stress at 4 °C for 24 h. The third unfolded rosette leaf was taken from each plant for NBT staining [21,36]. First, an NBT solution was prepared. A total of 0.05 g NBT and 0.5 mL 1 M phosphate buffer (pH = 7.8) was added into a 50 mL centrifuge tube, and ddH_2_O was added to make the solution reach 50 mL. The sampled leaves were then dyed in NBT solution for 0.5–1.0 h and taken out. The stained leaves were decolorized with 95% alcohol until the chlorophyll was completely degraded for easy observation and photography. Three replicates were performed for each sample.

### 4.9. Analysis of Expression Patterns of ROS-Related Marker Genes and Cold-Responsive Genes in Transgenic Plants

In order to study the response of *ZmDHN15* to cold, the expression patterns of ROS-related marker genes and cold-responsive genes in *Arabidopsis thaliana* were analyzed using *AtACTIN1* as an internal reference gene. Three replicates were performed for each sample, and all primers used in the experiments are listed in Supplementary Table S1.

### 4.10. Statistical Analysis

All results in this study were performed in more than three replicates. Data were expressed as the mean of triplicate values, and the error represented the SD(standard deviation). Statistical analyses and plotting were performed using GraphPad Prism 8 (V8.4.3, GraphPad, Changchun, China). The statistical significance of the difference was confirmed by a Student’s *t*-test; asterisks indicate statistically significant differences: *p* < 0.05 (*) and *p* < 0.01 (**).

## 5. Conclusions

In conclusion, *ZmDHN15* is a positive regulator of cold tolerance in yeast and Arabidopsis. According to the subcellular localization results, the ZmDHN15 fusion protein is localized in the cytoplasm. Under cold-stress conditions, the overexpression of *ZmDHN15* significantly improves the cold resistance of yeast. Subsequent analysis shows that *ZmDHN15* can promote the growth of Arabidopsis plants and accumulate less ROS. Furthermore, under cold-stress conditions, the overexpression of *ZmDHN15* in Arabidopsis can activate the expression of key genes in the ROS-signaling- and CBF-dependent pathways. Our findings advance our understanding of the function of DHN genes in maize.

## Data Availability

All data generated or analyzed during this study are available within.

## Supplementary Materials

The following supporting information can be downloaded at: https://www.mdpi.com/article/10.3390/ijms24010480/s1.
