# Supplementary material for: Overexpression of ZmDHN15 Enhances Cold Tolerance in Yeast and Arabidopsis
Source: Int J Mol Sci. 2022 Dec 28;24(1):480. doi: 10.3390/ijms24010480 (PMC9820458; doi:10.3390/ijms24010480)
Supplement: Supplementary file 1 [file ijms-24-00480-s001.zip › ijms-2080054-supplementary.pdf]

Supplementary Table S1. Primer sequences

Destination segment name	Primer sequences（5’-3’）
Q-ZmDHN1-F	CGAAGACTCGTTTAGGAGAAAC
Q-ZmDHN1-R	TGCTGACCGTACTCCATGATG
Q-ZmDHN2-F	AAGCCAGCCAAAGTTCAGAC
Q-ZmDHN2-R	TGGTAGTGCGCCATACTTCCT
Q-ZmDHN3-F	AAGCCAAAAGGCACTGAAGAAG
Q-ZmDHN3R	ACAGAACAGATCAGCAGGCTAGCTA
Q-ZmDHN4-F	TAAAGCCAGCCAAAGTTCAGA
Q-ZmDHN4-R	TGGTAGTGCGCCATACTTCCT
Q-ZmDHN15-F	AAGCCAAAAGGCACTGAAGAAG
Q-ZmDHN15-R	ACAGAACAGATCAGCAGGCTAGCTA
Q-ZmACTIN1-F	CCACCCTACAATCTTCTTTCCC
Q-ZmACTIN1-R	GCTTGATGTCTCCTGCCTCTTC
ZmDHN15-F	AAGGCACTGAAGAAGCCAGTCA
ZmDHN15-R	GAAACCAAAGCAATTATTAACGCAT
pYES2-ZmDHN15-F	CCCAAGCTTAAGGCACTGAAGAAGCCAGTCA
pYES2-ZmDHN15-R	CGCGGATCCGAAACCAAAGCAATTATTAACGCAT
pA7-ZmDHN15-F	ACGGGGGACTCTTGACCATGGAAGGCACTGAAGAAGCCAGTCA
pA7-ZmDHN15-R	TCTCCTTTACTAGTCAGATCTGAAACCAAAGCAATTATTAACGCAT
pCAMBIA3301-ZmDHN15-F	ACTCTTGACCATGGTAGATCTAAGGCACTGAAGAAGCCAGTCA
pCAMBIA3301-ZmDHN15-R	GGGAAATTCGAGCTGGTCACCGAAACCAAAGCAATTATTAACGCAT
Bar-F	TGACGCACAATCCCACTATCCT
Bar-R	GAAACCCACGTCATGCCAGT
Q-AtSOS1-F	TGTTGCGTCACTTTGGGTAT
Q-AtSOS1-R	TCCTCGT-CATCCCTTAGTTC
Q-AtSOD1-F	CAGCTTAGAGAGAGAGAGAG
Q-AtSOD1-R	CCATGTCATCCAAAGGGAGAA
Q-AtCAT3-F	GCCGAGAGAAGACTGTGATTAG
Q-AtCAT3-R	CGTCGACCCATCTGGTTATG
Q-AtCBF1-F	GGCTGCGATGGAGATGGAGA
Q-AtCBF1-R	GGGCATTGTCGGCGTTGTT
Q-AtCBF2-F	GCTCCGATTACGAGTCTCCG
Q-AtCBF2-R	CATCCCCAACATCGCCTCTT
Q-AtCBF3-F	GATGACGACGTATCGTTATGGA
Q-AtCBF3-R	TACACTCGTTTCTCAGTTTTACAA
Q-AtRD29A-F	AGCCAAAACAGAGCACTTACACA
Q-AtRD29A-R	AGCCAAAACAGAGCACTTACACA
Q-AtCOR15B-F	GAAGCCAATGAAACTGCGACT
Q-AtCOR15B-R	TACCCTCTACGAACTCAGCCG
Q-AtCOR47-F	ACCAACAAGAATGCCTTCCA
Q-AtCOR47-R	GCCGCATCCGATACACTCTTT
Q-AtACTIN1-F	CTTGAGGTAGGATGAGACTGAG
Q-AtACTIN1-R	CTGACTGAAGCAGATGATTCTG
